# 

**Published:** 1909-06

**Authors:** 


					K
JUNE, 1909.
7~?  . i i.
-.whl!N bLNtt~/U. "I- i
Vol. XXVII. x?^fo, 104.
the / Liu,i,Q>y^
BRISTOL V">-?)
MEDIGO-CHIRURGICAE
JOURNAL
^ PUBLISHED UNDER THE AUSPICES OF
THE BRISTOL MEDICO-CHIRURG1CAL SOCIETT.
Editor: R. SHINGLETON SMITH, M.D., B.Sc.
WITH WHOM ARK ASSOCIATED
J. MICHELL CLARKE, M.A., M.D.,
J. LACY FIRTH, M.S., M.D., JAMES SWAIN, M.S., M.D.
P. WATSON WILLIAMS, M.D., Assistant Editor.
Editorial Sicretary: J. A. NIXON, B.A., M.B.
BRISTOL: J. W. ARROWSMITH.
London : J. & A. Churchill, 7 Great Marlborough Street.
Price One Shilling and Sixpence.

				

